# Surface-enhanced Raman spectroscopy analysis of glucose in spent embryo culture medium: correlation with embryo developmental potential

**DOI:** 10.3389/fcell.2026.1884109

**Published:** 2026-07-15

**Authors:** MengHan Jin, Shan Huang, Jun Zhang, LingYin Kong, Tao Wang, QingKai Guo, Bo Liang, Jian Ou, QingXia Meng

**Affiliations:** 1 Center of Reproduction and Genetics, The Affiliated Suzhou Hospital of Nanjing Medical University, Suzhou Municipal Hospital, Gusu School, Nanjing Medical University, Suzhou, China; 2 Basecare Medical Device Co., Ltd., Suzhou, China; 3 State Key Laboratory of Microbial Metabolism, Joint International Research Laboratory of Metabolic and Developmental Sciences, School of Life Sciences and Biotechnology, Shanghai Jiao Tong University, Shanghai, China

**Keywords:** assisted reproductive technology, embryonic potential assessment, glucose, metabolites, surface-enhanced Raman spectroscopy

## Abstract

**Introduction:**

Accurate and non-invasive assessment of early embryonic metabolic biomarkers and their association with developmental potential remains a major challenge in assisted reproductive technology (ART). Glucose is a key metabolic component associated with embryo developmental competence, and its residual level in spent embryo culture medium (SECM) may reflect embryonic metabolic activity and provide valuable information for embryo assessment.

**Methods:**

In this study, surface-enhanced Raman spectroscopy (SERS) was used for the relative quantification of residual glucose in SECM. A mouse model was applied to compare residual glucose levels between blastocyst-forming and non-blastocyst-forming groups. In addition, quantitative SERS (qSERS) stratification based on an optimized threshold was performed to evaluate the association between residual glucose levels and blastocyst developmental outcomes.

**Results:**

The results demonstrated the feasibility and analytical performance of the SERS-based metabolic analysis, with an analytical repeatability relative standard deviation (RSD) of 1.22% and a regression goodness-of-fit *R* ^2^ of 0.996. In the mouse model, residual glucose levels differed significantly between the blastocyst-forming and non-blastocyst-forming groups, indicating that glucose utilization is associated with blastocyst formation. Furthermore, qSERS stratification using the optimized threshold supported the association between residual glucose levels and blastocyst developmental outcomes, with a mean accuracy of 69.13%.

**Discussion:**

These findings suggest that residual glucose in SECM is an informative extracellular metabolic feature for evaluating embryo developmental potential. However, the predictive capacity of glucose alone remains limited, and future embryo assessment should integrate additional metabolic or morphokinetic indicators for a more comprehensive evaluation. Overall, this SERS-based glucose sensing strategy provides a technical basis for future non-invasive and multi-parametric embryo assessment in ART.

## 1 Introduction

Approximately 15% of couples worldwide are affected by infertility, with etiologies encompassing tubal obstruction, ovulatory disorders, impaired sperm quality, endometriosis, among others (Saha et al., 2021). ART offers a systematic medical solution to these challenges. For instance, *in vitro* fertilization (IVF) directly addresses fertility barriers and plays a critical role for specific populations: young cancer patients can preserve fertility prior to gonadotoxic treatments through oocyte or sperm cryopreservation, while women over 35 years experiencing diminished ovarian reserve may achieve significantly higher pregnancy success rates *via* ART (Venkatesh et al., 2025). Consequently, the evaluation and selection of embryos with higher reproductive potential become of paramount importance during ART procedures.

Within the realm of IVF embryo developmental potential assessment, non-invasive evaluation methods based on metabolic profiling *via* spent embryo culture medium (SECM) have garnered significant expert interest (Alizadeh Moghadam Masouleh et al., 2025). As early as the 1967, John Biggers et al. elucidated fundamental metabolic patterns in embryonic cellular development (Biggers et al., 1967). Subsequent extensive research into the underlying mechanisms has progressively shaped the now established field of embryo metabolomics, providing a robust theoretical foundation for assessing developmental potential through metabolic activity (Sharpley et al., 2021; Chi et al., 2020). However, conventional metabolic analysis techniques such as mass spectrometry (Zhang et al., 2024) and nuclear magnetic resonance (NMR) spectroscopy (Cui et al., 2025) exhibit inherent limitations, including high operational costs, lack of standardization, and suboptimal analytical throughput. While conventional Raman spectroscopy has proven effective for qualitative analysis in previous studies (Liang et al., 2019; Zheng et al., 2021; Meng et al., 2023), it suffers from inadequate precision in quantitative assessments, particularly within complex biological systems where accurate quantification of specific com-ponents remains challenging.

In response to the challenges, it is of considerable significance to develop quantitative analytical techniques that combine non-invasiveness, specificity, and precision for accurately assessing embryonic metabolic status. Numerous biomarkers within metabolic pathways during early embryonic development are implicated in developmental potential evaluation (Deng et al., 2024), including glucose, pyruvate, lactate, amino acids, and even cell-free DNA (cfDNA). For instance, reported correlations between cellular activity and associated metabolites include glucose metabolism linked to glucose transporter membrane protein activity (Gao et al., 2020), lactate metabolism associated with oxygen activity (Yang et al., 2021), lipid metabolism related to cytoplasmic function (Li et al., 2023; He et al., 2025), and cfDNA associated with nuclear integrity (Scalici et al., 2014). Additional markers involve organelles and their activity indicators, such as cytochrome c for mitochondrial function (Song et al., 2025). In recent years, SERS-based specific analysis strategies have been extensively reported. These include the construction of SERS probes utilizing the specificity of bioenzymes. For example, employing glucose oxidase for targeted glucose recognition (Sun, 2022; Zhang et al., 2021; Chen et al., 2024). From a cell biology perspective, embryonic metabolic activity is closely related to developmental competence (Xu et al., 2025).

Surface-enhanced Raman spectroscopy (SERS) relies on localized surface plasmon resonance (LSPR) in metallic nanostructures to generate strong localized electromagnetic fields, which substantially enhance Raman scattering signals. This enables sensitive detection of trace-level analytes and addresses the inherently weak signals in conventional Raman spectroscopy (Ding et al., 2017). Building on these advantages, this study applies a SERS-based method to quantitatively evaluate the association between glucose metabolism and blastocyst formation potential in mouse embryos, supporting its potential as an emerging tool for assessing embryonic developmental physiology.

## 2 Materials and methods

### 2.1 Source

**FIGURE 1:**
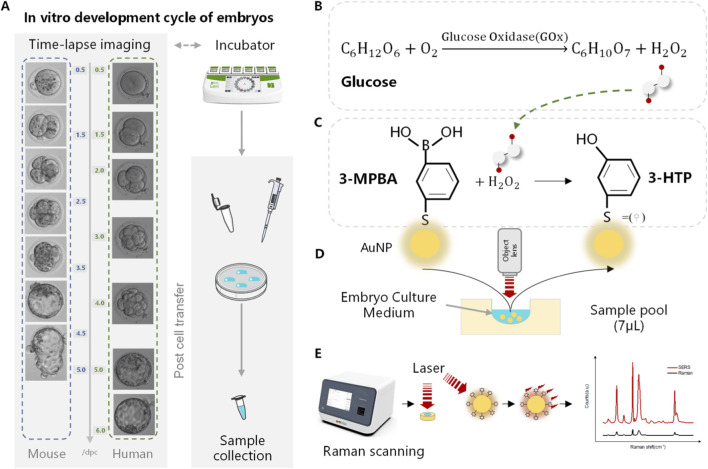
Experimental design and principle. **(A)** Schematic diagram of time-lapse microscopic imaging during early *in vitro* culture of embryos. **(B)** Reaction scheme of glucose oxidation catalyzed by glucose oxidase. **(C)** Reaction scheme of 3-MPBA with H_2_O_2_ forming 3-HTP. **(D)** Schematic of culture medium signal acquisition. **(E)** Diagram of Raman signal enhancement. (dpc: days post coitum).

IVF-derived embryos were cultured in a time-lapse incubator from the zygote to the blastocyst stage (Figure 1A). The animal use protocol involving mouse samples in this study was reviewed and approved by the Institutional Animal Care and Use Committee (IACUC) of GenePharma Co., Ltd. (Suzhou) under approval number 2022035. C57BL/6N mice (Beijing Vital River Laboratory Animal Technology Co., Ltd.) were superovulated by intraperitoneal injection of 7.15 IU pregnant mare serum gonadotropin (PMSG), followed 48 h later by 5 IU human chorionic gonadotropin (hCG). At 13–14 h post-hCG, females were euthanized by cervical dislocation, oocytes were retrieved from the oviductal ampullae, and sperm were collected from the cauda epididymis. IVF was subsequently performed, and embryos were cultured at 37 °C in 5% CO_2_. Multiple embryos were obtained from several female mice within the same IVF batch. Each embryo was cultured individually in an independent microdroplet, and spent embryo culture medium (SECM) was collected separately for qSERS analysis.

### 2.2 Samples

The experimental samples in this study comprised two parts: validation experiments using basic reagents and assays involving animal-derived samples. The validation samples included 5 mM glucose solution, standard mouse embryo culture medium (5.6 mM glucose), and serial dilutions of this medium yielding glucose concentrations ranging from 0.56 to 5.6 mM. In the animal experiment, 119 embryos were successfully fertilized. Among them, 87 developed into blastocysts (blastocyst-forming group), while the remaining 32 embryos arrested between day 0 and day 5 post coitum (non-blastocyst-forming group).

### 2.3 Reagents

3-Mercaptophenylboronic acid (3-MPBA, CAS: 352526–01–3, Shanghai yuanye Bio-Technology Co., Ltd.), glucose oxidase (GOx, ≥100 U/mg, CAS: 9001-37-0, Sigma-Aldrich), 60 nm spherical gold nanoparticles (AuNPs) colloidal solution (ABZW-1-60, Biotyscience, China), 7.5% hydrogen peroxide solution (Nanjing Chemical Reagent Co., Ltd.), Phosphate Buffered Saline (PBS) buffer (pH 7.4, prepared with NaBr solution), and ultrapure water were used. Glucose solutions and embryo culture media were supplied by Basecare Medical Device Co., Ltd., and samples of various concentrations were obtained through serial dilution. All reagents were of analytical grade and used without further purification.

### 2.4 Experimental

A mixture of 2 μL 1.5 mM 3MPBA and 998 μL 0.05 mg/mL AuNPs was incubated at room temperature for 2 h to form 3-MPBA@AuNPs conjugates, followed by three centrifugation cycles (4,000 rpm, 5 min). The pellet was resuspended in 438 μL ultrapure water and stored at 4 °C. For assays, 15 μL of 3-MPBA@AuNPs was mixed with 0.75 μL culture medium, 4.75 μL PBS, and 2 μL glucose oxidase, then incubated at 37 °C for 90 min (Figures 1B,C). The enzymatically reacted 3-HTP@AuNPs solution was subsequently analyzed by SERS to obtain Raman spectra (Figures 1D,E). Each SERS measurement was acquired with an exposure time of 90 s, and the final spectrum was obtained by averaging 3 accumulations.

### 2.5 Equipments

The experimental setup included a Raman spectrometer (BaseRaman, China), as depicted in Figure 1E, equipped with a 785 nm semiconductor laser with a maximum output power of 495 mW and approximately 300 mW at the focal point. A fiberoptic probe with a working distance of 5.4 mm and a spectral range of approximately 200–3,200 cm^-1^ was used. The instrument was calibrated using a single-crystal silicon reference, showing a characteristic peak at 520.5 ± 0.3 cm^-1^. Additional equipment consisted of electronic analytical balance (JA10003B, China), vortex mixer (VORTEX-6, China), centrifuge (Mini-7K, China), and constant temperature metal bath (HH-1, China).

### 2.6 Preprocessing

The preprocessing of the collected Raman spectra involved spectral range cropping, smoothing, baseline correction, and normalization. The fingerprint region of 600–1800 cm^-1^ was selected for analysis. Spectral noise was reduced by smoothing with the Savitzky-Golay algorithm. Baseline correction was then performed by fitting the background using the Statistics-sensitive Nonlinear Iterative Peak-clipping (SNIP) algorithm and sub-tracting it. Finally, normalization was applied to scale the spectral intensity to the range of [0, 1], ensuring uniform data intensity across all samples.

### 2.7 Methodology

A quantitative surface-enhanced Raman spectroscopy (qSERS) approach was employed for the relative quantification of glucose. Rather than directly detecting glucose molecules, this method indirectly reflects the relative glucose concentration by monitoring the SERS spectral changes associated with the H_2_O_2_-mediated oxidation of the Raman probe 3-MPBA into 3-HTP. The measured glucose level was defined as residual glucose, referring to the amount of glucose remaining in the SECM after embryo incubation. Specifically, a calibration (regression) model was constructed using SERS spectra acquired from a series of glucose standards with varying concentrations. The intensity ratio of two characteristic Raman bands, $I_{{881cm}^{-1}}/I_{{997cm}^{-1}}$ , was used as the independent variable to mitigate variability introduced by absolute intensity fluctuations (e.g., laser power drift, substrate heterogeneity, and focus differences), whereas the nominal glucose concentration served as the dependent variable. Model performance was evaluated using the coefficient of determination (*R* ^2^) and the adjusted *R* ^2^ (adj. *R* ^2^) to assess goodness of fit. Reproducibility of the measurements was quantified by the relative standard deviation (RSD) of the ratio metric across replicate spectra. Statistical significance between the blastocyst-forming and non-blastocyst-forming groups was assessed using Welch’s t-test. Student’s t-test was additionally performed as a sensitivity analysis to confirm the consistency of the statistical conclusion, with p < 0.05 considered statistically significant.

### 2.8 Software

The raw Raman spectra underwent a preprocessing pipeline encompassing spectral cropping, smoothing, baseline correction, and normalization, which was implemented in BaseRamanSPEC software (v2.5.9). All subsequent data analysis, statistical computation, and visualization were conducted using R (v4.1.0), Python (v3.6.13), and Origin 2024, supplemented by the SciPy (v1.5.4) and Matplotlib (v3.3.4) libraries.

## 3 Results

### 3.1 Evaluating the effects of enzyme presence, reaction time, and nanoparticle size using glucose solution

**FIGURE 2:**
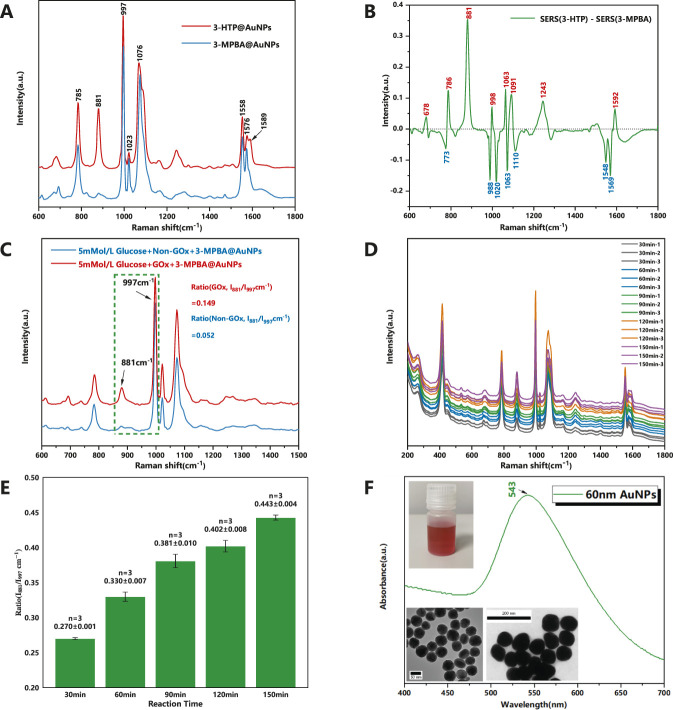
**(A)** SERS of 3-MPBA@AuNPs and 3-HTP@AuNPs. **(B)** Difference spectrum obtained by subtracting the 3-MPBA@AuNPs signal from that of 3-HTP@AuNPs. **(C)** Control experiment comparing SERS signals with and without enzymatic catalysis in 5 mM glucose solution. **(D)** Raw SERS illustrating the time-dependent formation of 3-HTP at different enzymatic reaction time points. **(E)** Bar chart of SERS quantification results for 5 mM glucose solution samples across enzymatic reaction times of 30–150 min **(F)** UV-Vis absorption spectrum, TEM image, and physical photograph of 60-nm diameter AuNPs.

The unique SERS “fingerprint” enables trace-level and accurate detection of target molecules. As shown in Figure 2A and Supplementary Table S1, a comparison was made between the SERS signals of 3-MPBA and 3-HTP immobilized on colloidal gold nanoparticles. The main characteristic peaks of 3-MPBA include 785 cm^-1^ (C-H out-of-plane bending), 997 cm^-1^ (C-C in-plane bending), 1,023 cm^-1^ (C-H in-plane bending), 1,076 cm^-1^ (C-C in-plane bending coupled with C-S stretching), 1,558 cm^-1^ (non-totally symmetric ring stretching), and 1,576 cm^-1^ (totally symmetric ring stretching). In contrast, 3-HTP exhibits distinct characteristic peaks around 881 cm^-1^ (ring stretching) and 1,589 cm^-1^ (totally symmetric ring stretching), which differ from those of 3-MPBA and confirm its Raman specificity (Liu et al., 2020). When 3-MPBA acts as a reducing agent and is oxidized by H_2_O_2_ to form 3-HTP, this reaction demonstrates that 3-MPBA can serve as a Raman probe for the accurate quantification of H_2_O_2_ concentration. Figure 2B presents the difference between the normalized spectra of 3-HTP@AuNPs and 3-MPBA@AuNPs, with the dominant characteristic band appearing at approximately 881 cm^-1^, this band can be indirectly used as a key response feature for glucose detection in the tested samples.

To validate the key functional specificity of GOx, a standard glucose solution (5 mM) was analyzed with or without the addition of the 3-MPBA@AuNPs/GOx system (Figure 2C). Raman spectra revealed a striking difference between groups, the control (without GOx) showed a peak intensity ratio of 0.052, serving as a baseline, whereas the experimental group (with GOx) exhibited a markedly higher ratio of 0.149. The increase confirmed the enzymatic specificity of GOx toward glucose, demonstrating its capacity to selectively identify target molecules in complex mixtures.

Accordingly, a mixture of NaBr and PBS buffer (pH 7.4) was employed during sample preparation to optimize detection efficiency (Peng et al., 2022). Under physiological conditions (37 °C, pH 7.4), GOx catalyzes the oxidation of glucose in the presence of oxygen, producing hydrogen peroxide (H_2_O_2_) and gluconic acid. In this system, 3-MPBA functions as a Raman probe for the specific detection of H_2_O_2_, thereby enabling the indirect quantification of glucose in solution. Figure 2D displays the SERS collected at different time points during the enzymatic reaction. In all reaction time groups, the peak at 997 cm^-1^ consistently exhibited the highest intensity, supporting its use as an internal reference. Meanwhile, the intensity of the peak at 881 cm^-1^ increased with prolonged reaction time, and the ratio of the two peak intensities showed a linear relationship (Figure 2E). At each time point, the standard deviation of the ratio values across replicate samples was ≤0.01, confirming high reproducibility and experimental stability. The UV-Vis absorption spectrum (Figure 2F) exhibits a distinct surface plasmon resonance (SPR) band centered at approximately 543 nm, which is characteristic of spherical AuNPs with a diameter around 60 nm. Figure 2F also includes TEM images that confirm the spherical shape and homogeneous size distribution of the synthesized AuNPs.

**FIGURE 3:**
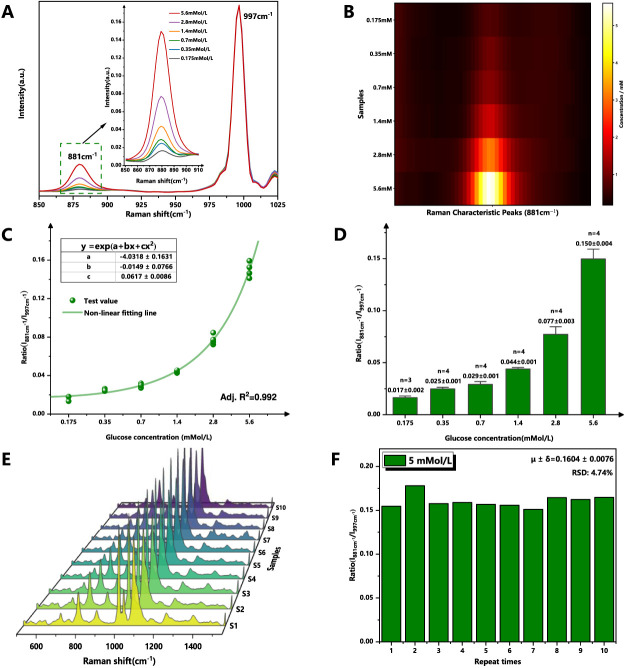
**(A)** SERS spectra (850–1,025 cm^-1^) of glucose solutions at concentrations of 0.175–5.6 mM. **(B)** Heatmap of the Raman intensity at 881 cm^-1^, showing the glucose concentration gradient across the solution. **(C)** Nonlinear calibration curve correlating the SERS signal ratio with glucose concentration. **(D)** SERS-based quantification of glucose across a concentration gradient. (n: sample number) **(E)** SERS from ten replicate measurements of 5 mM glucose solution. (S1-S10: Sample1-10) **(F)** Relative standard deviation (RSD) values were calculated from ten replicate measurements of a 5 mM glucose solution.

The Raman spectral region between 850 and 1,025 cm^-1^ was analyzed (Figure 3A), revealing two characteristic peaks at 881 cm^-1^ and 997 cm^-1^. The intensity of the 881 cm^-1^ peak exhibited a positive correlation with glucose concentration (Figure 3B). Using glucose standards at varying concentrations, the corresponding intensity ratios were calculated and are summarized in Figure 3C, which demonstrates a nonlinear relationship with glucose concentration. Nonlinear regression analysis (Figure 3D) produced a fitting equation with an adjusted *R* ^2^ of 0.992. To evaluate reproducibility, ten replicate measurements (S1-S10) were performed on 5 mM glucose solution samples, the raw SERS data are shown in Figure 3E. The histogram of the corresponding SERS quantification ratios is presented in Figure 3F, yielding a mean ratio of 0.1604 ± 0.0076 and a relative standard deviation (RSD) of 4.74%, which meets the acceptance criterion of RSD <5%. These results confirm the specificity and reproducibility of the proposed SERS-based glucose detection method.

### 3.2 Experimental condition optimization and application to embryo culture medium

**FIGURE 4:**
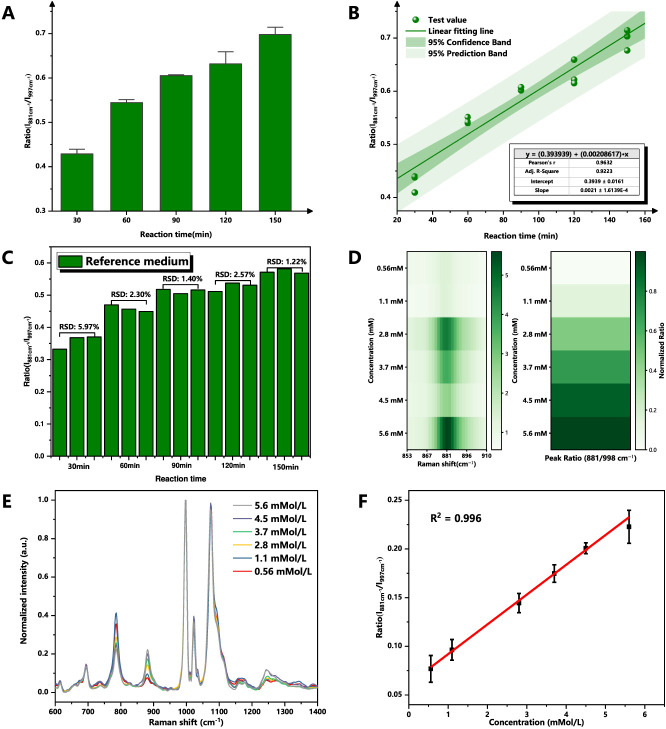
**(A)** Simulated time-dependent concentration profiles for the H_2_O_2_-driven oxidation of 3-MPBA to 3-HTP over 30–150 min. **(B)** Linear calibration curve correlating the SERS signal ratio with different reaction time. **(C)** Relative standard deviation (RSD) values were calculated from ten replicate qSERS-based glucose measurements performed in a reference culture medium. **(D)** Heatmaps of the SERS signal around 881 ± 30 cm^-1^ and the corresponding intensity ratio $I_{{881cm}^{-1}}/I_{{997cm}^{-1}}$ for culture medium samples with glucose concentrations ranging from 0.56 to 5.6 mM. **(E)** Quantification of glucose concentrations at different dilutions using SERS spectra in the 600–1,400 cm^-1^ region. **(F)** Linear regression fitting for glucose concentration in embryo culture medium.

To further optimize the experimental, factors potentially affecting the accuracy and stability of the results were considered, including the enzymatic reaction time during Raman probe preparation and the particle size of the spherical gold colloidal nanoparticles used. As shown in Figure 4A, quantitative analysis in the form of bar and box plots is presented for enzymatic reaction time points at 30, 60, 90, 120, and 150 min. The results indicate a continuous increase in the Ratio value over enzymatic reaction time, reflecting the slow reaction process of H_2_O_2_ generation, with an overall linearly increasing trend. Figure 4B displays the linear regression fitting curve for different enzymatic reaction durations. The adjusted *R* ^2^ of this linear model was 0.92, with a correlation coefficient of 0.96. Further validation of reproducibility across different reaction times is shown in Figure 4C. For each time group, 3 replicate samples were analyzed, the Ratio values were collected and the RSD was calculated. The results demonstrate that the 150-min group had the lowest RSD (1.22%), followed by the 90-min group with an RSD of 1.4%.

Under the optimized conditions, validation experiments were performed using mouse embryo culture medium. As shown in Figure 4D, the left panel presents a heatmap illustrating the SERS signal distribution within the spectral region of approximately 881 ± 30 cm^-1^ for glucose concentrations ranging from 0.56 to 5.6 mM (specifically 0.56, 1.1, 2.8, 3.7, 4.5, and 5.6 mM). The results indicate that relying solely on the 881 cm^-1^ characteristic band of 3-HTP, generated from the Raman probe, is insufficient for accurate relative quantification of glucose concentration, as the signal change in this region alone does not exhibit a sufficiently robust concentration-dependent trend. Therefore, as shown in the right panel, the intensity ratio between the 881 cm^-1^ characteristic peak and the 997 cm^-1^ internal standard peak was calculated and used as the quantitative metric. By incorporating the internal standard, the ratio-based parameter $I_{{881cm}^{-1}}/I_{{997cm}^{-1}}$ , provides a more reliable basis for the relative quantification of glucose concentration. Serial dilution was used to prepare medium samples at these six specified concentrations. The corresponding qSERS results are summarized in Figures 4E,F. A linear relationship was established between glucose concentration and the quantitative SERS signal, represented by the intensity ratio of the characteristic peaks at 881 and 997 cm^-1^, with the regression model yielding a coefficient of determination (*R* ^2^) of 0.996. Notably, the established detection range of 0.56–5.6 mM covers the biologically relevant glucose concentrations in embryo culture systems. Previous studies have shown that glucose concentrations in blastocyst-stage culture media are typically around 2.5–3.3 mM (Toporcerová et al., 2025), and that human preimplantation embryos exhibit measurable glucose uptake, the variation of which is associated with blastocyst formation and blastocyst quality. Therefore, this detection range is sufficient to cover the basal glucose levels in D5-D6 culture media as well as the associated biologically relevant fluctuations.

**FIGURE 5:**
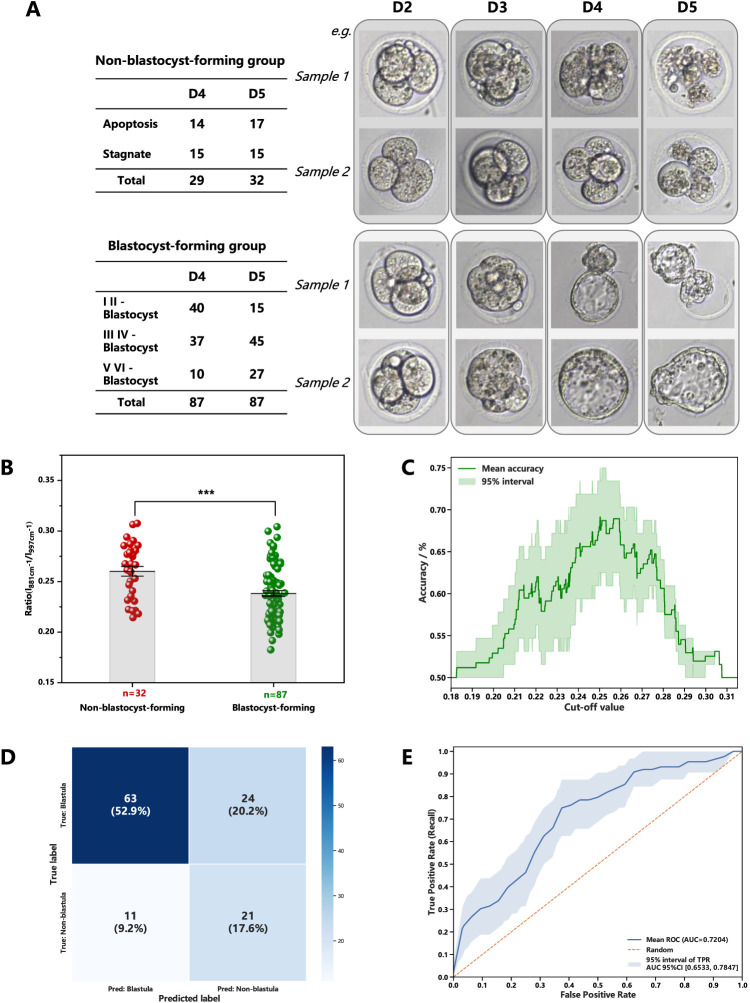
**(A)** Records from the non-blastocyst-forming and blastocyst-forming groups, along with corresponding bright-field micrographs from D2 to D5 (e.g., Sample one to two of each group; see Figure A2 for additional samples). D2-5 denotes days post-fertilization. **(B)** Comparison of glucose levels via qSERS in arrested embryos (n = 32) and blastocysts (n = 87) cultured in D5 medium (Welch’s t-test and Student’s t-test; ***p < 0.001). **(C)** Cut-off optimization for qSERS-based classification of blastocyst formation outcomes (1,000 resampling iterations). **(D)** Confusion matrix for prediction of blastocyst formation outcome at the cut-off value of 0.2502. **(E)** ROC curve and corresponding AUC for prediction of blastocyst formation outcome at the cut-off value of 0.2502.

As shown in Figure 5A, Supplementary Figure S2, morphological images of individual embryos from both outcome groups were recorded at daily developmental stages to monitor embryonic progression. In the non-blastocyst-forming group on D5, embryos exhibited either developmental arrest (15 samples, e.g., Sample 2, Sample 3) or apoptosis (17 samples, e.g., Sample 1, Sample 5). In contrast, blastocysts in the blastocyst-forming group were classified into stages I-VI according to standard developmental chronology, with 15 samples at early stages (I-II), 45 samples at intermediate stages (III-IV), and 27 samples at advanced stages (V-VI).

Supplementary Figure S1 presents a quantitative comparison of residual glucose levels in D5 culture media between the blastocyst-forming group (n = 87) and the non-blastocyst-forming group (n = 32) using qSERS. As summarized in Figure 5B and Table 1, the two groups showed a statistically significant difference in residual glucose levels based on Welch’s t-test (p = 2.23 × 10^−4^), and this result was consistent with Student’s t-test as a sensitivity analysis (p = 1.27 × 10^−4^). The between-group effect size was Cohen’s d = 0.814, with a 95% confidence interval of 0.399–1.236, indicating a moderate-to-large difference in residual glucose levels between the non-blastocyst-forming and blastocyst-forming groups. Specifically, the blastocyst-forming group exhibited lower residual glucose levels (mean = 0.238, median = 0.240) than the non-blastocyst-forming group (mean = 0.261, median = 0.265), which is consistent with greater cumulative glucose utilization by embryos that progressed to the blastocyst stage. Although some dispersion was observed in both groups, the non-blastocyst-forming group showed greater variability (SD = 0.028, IQR = 0.043) than the blastocyst-forming group (SD = 0.027, IQR = 0.030), suggesting a more heterogeneous extracellular metabolic profile among embryos with impaired developmental outcomes. Together, these results indicate that qSERS-derived residual glucose levels are associated with blastocyst developmental outcomes.

**TABLE 1:** Summary of Analysis between non-blastocyst-forming group and blastocyst-forming group.

Metrics	Blastocyst-forming	Non-blastocyst-forming
Sample size	87	32
Mean	0.238	0.261
Standard deviation	0.0272	0.0282
Median	0.240	0.265
Minimum	0.18	0.21
Maximum	0.30	0.31
Interquartile range	0.0300	0.0425
Normality (p)	0.107	0.116
Equal variance (p)	0.542	
p value	<0.001	
Cohen’s d	0.814 (95% CI: 0.399–1.236)	

As shown in Figure 5C, the SERS-based quantitative glucose metric was retrospectively evaluated in 119 samples. The analysis compared qSERS values from 32 non-blastocyst-forming embryos and 32 blastocyst-forming embryos (randomly selected from 87 blastocyst-forming cases). Classification accuracy was computed across cut-off values within 0.18–0.315 (step size = 0.0001). Random subsampling of the blastocyst-forming group was repeated for 1,000 iterations to obtain a robust performance estimate. The optimal performance was achieved at a cut-off of 0.2502, yielding a mean accuracy of 69.13% (SD = 0.0316) and a 95% interval of 0.6250–0.75. Using this optimized cut-off, the classification performance was further evaluated in the full dataset of 119 samples. As shown in Figure 5D, the corresponding confusion matrix yielded 63 true positives, 24 false negatives, 21 true negatives, and 11 false positives, corresponding to an overall accuracy of 70.6%, a sensitivity of 72.4%, and a specificity of 65.6%. Consistently, ROC analysis (Figure 5E) showed an AUC of 0.7204, with a 95% confidence interval of 0.6533–0.7847.

These results indicate that blastocyst development is associated with higher energy demand, and that active glucose consumption may reflect embryonic metabolic activity rather than serve as a definitive biomarker of embryo health. Therefore, assessment of embryo developmental potential should not rely on glucose alone, but should be further integrated with metabolic levels of additional biomarkers, such as pyruvate, lactate, amino acids, and lipid-related metabolites, to provide a more comprehensive evaluation of embryonic competence.

## 4 Discussion

Non-invasive assessment of embryo developmental competence aims to identify informative biomarkers in spent embryo culture medium (SECM) during *in vitro* culture. Candidate biomarker classes include metabolites, extracellular vesicles, proteins, and multiple nucleic acid species, such as cell-free DNA, mitochondrial DNA, and small RNAs (Zagers et al., 2025). Among these, metabolomics is particularly attractive because it captures functional readouts of embryo–microenvironment exchange without disturbing development. From the perspective of cellular endocrinology, these metabolic readouts should not be regarded merely as passive indicators of nutrient consumption, but may also reflect downstream effects of endocrine and paracrine signaling. Recent evidence indicates that preimplantation embryo metabolism is tightly coupled to signaling networks including insulin/IGF-associated PI3K-AKT-mTOR, AMPK, and TGF-β/Nodal pathways, which collectively regulate nutrient uptake, mitochondrial activity, pluripotency maintenance, and lineage specification (Xu et al., 2025; Lee and Rinaudo, 2024). Metabolic profiling typically focuses on energy substrates, such as glucose, pyruvate, and lactate, amino acid turnover, lipid metabolism, and related intermediates (Supplementary Figure S3), thereby providing quantitative features that can support embryo assessment (Alizadeh Moghadam Masouleh et al., 2025).

Against this background, residual glucose in D5 SECM was selected in the present study as a representative extracellular metabolic readout to evaluate whether glucose utilization is associated with blastocyst formation. The blastocyst-forming group showed lower residual glucose levels than the non-blastocyst-forming group, suggesting that embryos progressing to the blastocyst stage may have higher cumulative glucose consumption during late preimplantation development. This interpretation is consistent with previous studies showing that preimplantation embryo development is accompanied by dynamic metabolic regulation and that glucose-related pathways are involved in developmental progression and lineage-associated metabolic remodeling (Alizadeh Moghadam Masouleh et al., 2025; Sharpley et al., 2021; Chi et al., 2020; Xu et al., 2025; Lee and Rinaudo, 2024). Therefore, the glucose-related qSERS signal may be associated, at least in part, with the metabolic transition accompanying progression from cleavage-stage embryos to blastocysts.

However, this association should be interpreted within the methodological and biological boundaries of SECM analysis. Residual glucose in SECM represents an indirect extracellular readout rather than a direct measurement of intracellular glucose transport, glycolytic flux, or mitochondrial metabolism. The interpretation of D5 glucose levels should therefore consider the developmental history of each embryo. Embryos that arrest before D5 may have shorter periods of active nutrient utilization, whereas embryos that retain partial metabolic activity before degeneration may leave different residual glucose profiles. In addition, apoptosis-like degeneration, severe fragmentation, or collapse-like changes may alter membrane integrity and the culture microenvironment, further affecting extracellular metabolite levels. In the present study, arrested or degenerating embryos were identified by routine morphological assessment, including developmental delay or arrest, severe fragmentation, blastomere degeneration, and collapse-like changes. These embryos were included in the non-blastocyst-forming group because they represent clinically relevant unsuccessful developmental outcomes, but their qSERS profiles should be interpreted as cumulative extracellular phenotypes rather than direct measurements of active metabolism at D5.

This interpretative framework also helps explain the greater variability observed in the non-blastocyst-forming group. The broader distribution of qSERS values may arise from asynchronous developmental arrest, differences in residual metabolic activity, and variable degrees of degeneration. Some embryos may arrest early with limited subsequent glucose consumption, whereas others may maintain partial metabolic activity before failing to form blastocysts. Degenerating embryos may further increase variability through altered membrane permeability or degradation-related changes in the culture medium. Thus, the dispersion of qSERS values in the non-blastocyst-forming group likely reflects multiple impaired developmental trajectories converging into the same morphological endpoint of failed blastocyst formation.

These findings are consistent with a growing body of evidence linking metabolic state to embryo development *in vitro*. Nami et al. reported altered concentrations of glucose, pyruvate, and lactate and ionic imbalance in SECM from patients with recurrent implantation failure, suggesting that dysregulated energy metabolism and ion homeostasis may compromise implantation potential (Nami et al., 2024). Beyond energy metabolism, amino acid turnover contributes carbon and nitrogen sources and participates in signaling. Elsayed et al. integrated embryo morphokinetics with SECM amino acid consumption profiles and reported improved discrimination for embryo selection using combined analysis (Adel et al., 2025). In parallel, advances in spectroscopic techniques have expanded the feasibility of rapid biochemical profiling across reproductive biofluids, including SECM (Wamaitha et al., 2020). From an endocrine perspective, glucose, pyruvate, and lactate are key metabolic nodes that may reflect hormone- and growth factor-regulated metabolic activity. Human embryo and stem cell studies have identified insulin and IGF1 receptor expression together with active PI3K/AKT/mTOR signaling in the blastocyst context, while recent mammalian evidence further supports an essential role for IGF1R-dependent signaling during blastocyst formation (Park et al., 2026; Noli et al., 2020). In addition, thyroid hormone T3 has been shown to stimulate mitochondrial replication and promote a shift from glycolysis toward oxidative phosphorylation in human preimplantation embryos, directly linking endocrine cues to measurable metabolic remodeling (Sui et al., 2025).

At the pathway level, embryo metabolism integrates multiple substrate classes rather than relying on a single nutrient. As schematized in Supplementary Figure S3a, glucose, amino acid, and lipid utilization are connected through central pathways such as glycolysis and the tricarboxylic acid (TCA) cycle, while amino acid catabolism links nitrogen handling to urea-cycle-related processes. Fluctuations in metabolite levels therefore reflect adaptive responses to nutrient availability and energetic demand. In this study, glucose was selected as a representative metabolite for experimental validation and for illustrating pathway-informed analysis. This choice is also supported by its position downstream of growth factor- and hormone-sensitive signaling pathways that regulate transporter activity, mitochondrial function, and biosynthetic state. Accordingly, glucose in SECM may serve not only as a marker of metabolic activity, but also as a candidate non-invasive readout related to embryo endocrine–metabolic interactions at the embryo–microenvironment interface.

Consistent with this pathway-level complexity, the classification performance for blastocyst developmental outcomes based on the optimized threshold suggests that residual glucose captures only part of the metabolic phenotype associated with embryo development. The observed AUC and accuracy indicate that glucose is informative, but not sufficient as an independent embryo selection biomarker. This is consistent with the concept that embryo competence is shaped by coordinated changes across multiple metabolic pathways, including glucose, pyruvate, lactate, amino acid, lipid, and mitochondrial metabolism. Therefore, the present findings should be interpreted as evidence of an association between residual glucose and blastocyst formation, rather than as support for glucose as a standalone predictor of developmental competence. A more informative strategy may be to integrate glucose with additional metabolites and time-lapse morphokinetic parameters, thereby capturing both biochemical and developmental dynamics. This interpretation is consistent with studies showing that combined metabolic or metabolomic features provide greater embryo assessment value than single analytes (Alizadeh Moghadam Masouleh et al., 2025; Nami et al., 2024; Adel et al., 2025; Tong et al., 2026).

From a technical perspective, selective and reproducible SERS detection depends on probe and substrate engineering, including high-affinity recognition elements, blocking strategies to reduce nonspecific adsorption, stringent washing, matrix-aware sample preprocessing, and internal standards to correct signal drift caused by laser-power variation or substrate heterogeneity (Markina and Markin, 2024; Geng et al., 2024; Bailey et al., 2016; Zhou G. et al., 2025; Bi et al., 2025; Zhou J. et al., 2025; Cheng et al., 2023). Robust spectral feature extraction and modeling can further improve analysis in complex matrices (Kamran et al., 2024; Yan et al., 2023). In this context, the specific contribution of the present study is that it provides a targeted, enzyme-mediated SERS strategy for quantifying a biologically relevant metabolite in microliter-scale SECM. Rather than replacing established morphological or morphokinetic assessment, this approach may complement existing embryo evaluation methods by adding a quantitative extracellular metabolic dimension.

Several methodological considerations should be noted when interpreting these results. The classification threshold was derived from the current dataset and should be considered exploratory until validated in independent cohorts. In addition, the present analysis compared blastocyst-forming and non-blastocyst-forming embryos, but did not include a sufficiently powered subgroup analysis based on detailed inner cell mass and trophectoderm grading. Therefore, the current results mainly support an association with blastocyst formation, while further studies are needed to determine whether glucose utilization differs between high- and low-quality blastocysts. Potential clustering related to embryos derived from multiple female mice within the same IVF batch should also be addressed in larger studies using appropriate statistical models.

For translation to human IVF, these methodological issues are compounded by additional clinical and operational challenges. Human embryo metabolism is influenced by inter-patient variability, including maternal age, infertility etiology, ovarian stimulation protocol, sperm-related factors, and culture conditions. Commercial culture media also differ in baseline glucose concentration and biochemical composition, which may affect residual metabolite measurements and require medium-specific calibration or normalization (Zagers et al., 2025). Clinical implementation would require minimal sample consumption, rapid turnaround, standardized sample handling, reproducible spectral acquisition, and compatibility with routine embryology workflows. If applied as an embryo selection tool, the approach would further require prospective validation, inter-laboratory reproducibility assessment, and regulatory evaluation to establish safety, reliability, and clinical utility.

Overall, the present study supports the feasibility of qSERS-based glucose analysis as a non-invasive extracellular metabolic readout in embryo culture medium. The findings suggest that residual glucose is associated with blastocyst formation, but its biological interpretation should be integrated with developmental timing, embryo morphology, and additional metabolic indicators. Future work using larger datasets, multi-metabolite panels, morphokinetic integration, and independent validation will be essential for developing robust SERS-based metabolic assessment strategies for assisted reproduction.

## 5 Conclusion

This study demonstrates that specificity-enhanced SERS enables relative quantification of residual glucose in SECM, providing a feasible strategy for non-invasive assessment of embryonic metabolic status. The blastocyst-forming group showed lower residual glucose levels than the non-blastocyst-forming group, indicating that increased glucose utilization is associated with blastocyst formation and may reflect metabolic remodeling during preimplantation development. The assay showed reliable analytical performance in SECM, with a regression goodness-of-fit of *R* ^2^ = 0.996, an analytical repeatability RSD of 1.22%. qSERS stratification based on the optimized threshold further supported an association between residual glucose and blastocyst developmental outcomes, while indicating that glucose should be integrated with additional metabolic or morphokinetic indicators. Overall, this SERS-based glucose sensing strategy provides a technical basis for non-invasive, multi-parametric embryo assessment in assisted reproduction and offers a foundation for further expanding metabolic biomarker panels and optimizing embryo assessment models.

## Data Availability

The raw data supporting the conclusions of this article will be made available by the authors, without undue reservation.

## Supplementary material

Supplementary Table S1 Assignment of Raman Vibrational Modes of 3-MPBA and 3-HTP (Saha et al., 2021).

Supplementary Figure 1 Comparative analysis of the SERS glucose ratio (I_881_/I_997_ cm^-1^) between embryo groups. Histograms display the ratio distribution in **(a)** blastocyst-forming (n = 87, *S1-S87) and **(b)** non-blastocyst-forming (n = 32, *S1-S32) groups.

Supplementary Figure 2 Records from the blastocyst-forming group and non-blastocyst-forming groups, along with corresponding bright-field micrographs from D2 to D5 (e.g., Sample 3–6). Records from the **(a)** blastocyst-forming group and **(b)** non-blastocyst-forming groups, along with corresponding bright-field micrographs from D2 to D5 (e.g., Sample 3–6). *D2-5, days post fertilization.

Supplementary Figure 3 Schematic illustration of the metabolic framework and the SERS-based monitoring strategy. **(a)** Glucose and Amino Acids Drive Macromolecule Synthesis. (The red dashed box indicates potential biomarkers within the glucose and amino acid metabolic pathways that be can used to assess embryonic developmental quality. tRNA: Transfer RNA; ATP: Adenosine Triphosphate; AMP: Adenosine Monophosphate; PP ∼ i∼: Pyrophosphate; OAA: Oxaloacetate; α-KG: α-Ketoglutarate.) **(b)** Functional mechanisms of multiple metabolites (e.g., glucose, amino acids) involved in the proposed research strategy during embryonic development. (Venkatesh et al., 2025; Alizadeh Moghadam Masouleh et al., 2025; Biggers et al., 1967; Sharpley et al., 2021; Chi et al., 2020; Zhang et al., 2024; Cui et al., 2025).
