# Supplementary material for: Surface-enhanced Raman spectroscopy analysis of glucose in spent embryo culture medium: correlation with embryo developmental potential
Source: Front Cell Dev Biol. 2026 Jul 15;14:1884109. doi: 10.3389/fcell.2026.1884109 (PMC13415918; doi:10.3389/fcell.2026.1884109)
Supplement: Supplementary file 1 [file DataSheet1.pdf]

Supplementary Material

Consents

Table S1. Assignment of Raman Vibrational Modes of 3-MPBA and 3-HTP

Figure S1. Comparative analysis of the SERS glucose ratio (I_{881}/I_{997} cm^{-1}) between embryo groups.

Figure S2. Records from the blastocyst-forming group and non-blastocyst-forming groups, along with corresponding bright-field micrographs from D2 to D5 (e.g., Sample 3-6.).

Figure S3. Schematic illustration of the metabolic framework and the SERS-based monitoring strategy.

Table S1. Assignment of Raman Vibrational Modes of 3-MPBA and 3-HTP [1]

Index	Raman shift(cm^{-1})	Vibration mode
1	785	C-H out-of-plane bending
2	881	Ring stretching
3	997	C-C in-plane bending
4	1023	C-H in-plane bending
5	1076	C-C in-plane bending coupled with C-S stretching
6	1558	Non-totally symmetric ring stretching
7	1576	Totally symmetric ring stretching
8	1589	Totally symmetric ring stretching

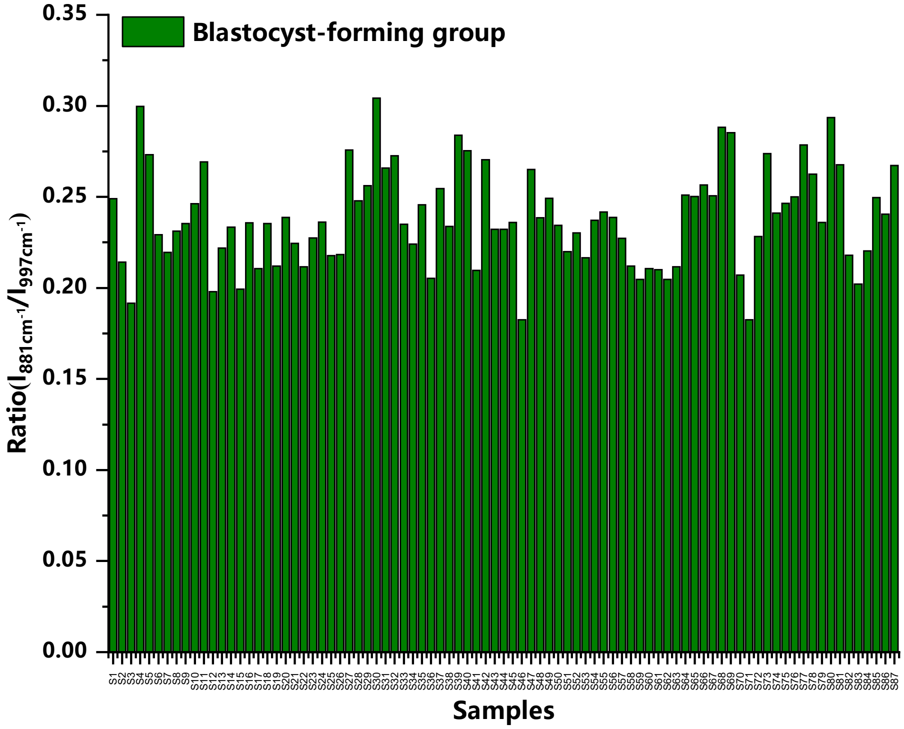

(a)

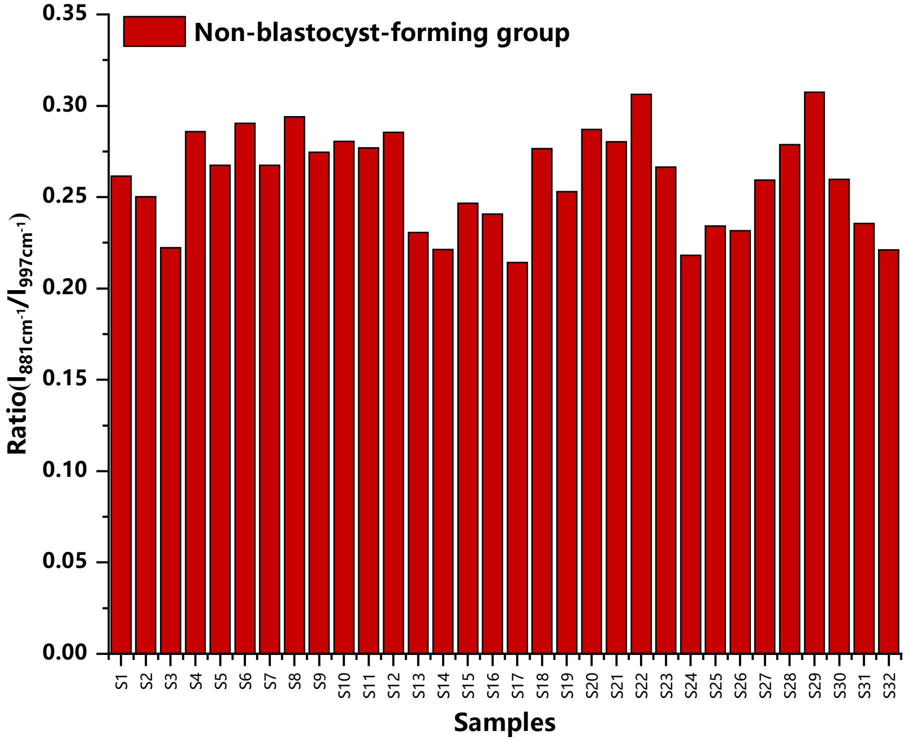

(b)

Supplementary Figure 1. Histograms display the ratio distribution in (a) blastocyst-forming (n = 87, *S1-S87) and (b) non-blastocyst-forming (n = 32, *S1-S32) groups.

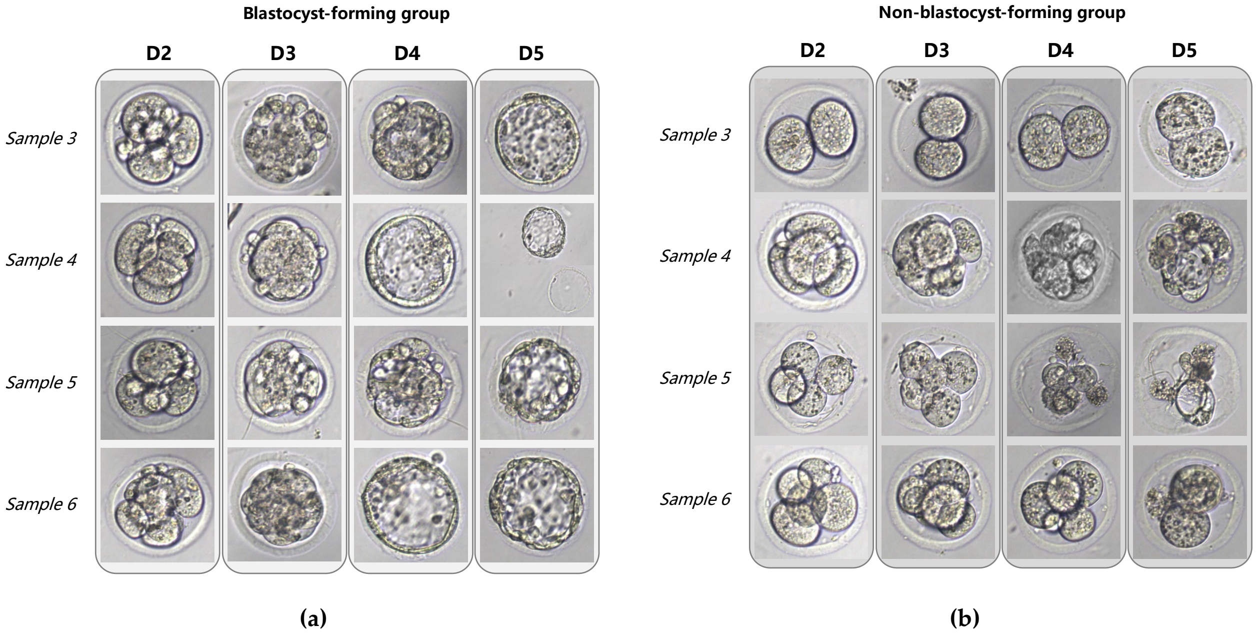

Supplementary Figure 2. Records from the (a) blastocyst-forming group and (b) non-blastocyst-forming groups, along with corresponding bright-field micrographs from D2 to D5 (e.g., Sample 3-6.). *D2-5, days post fertilization.

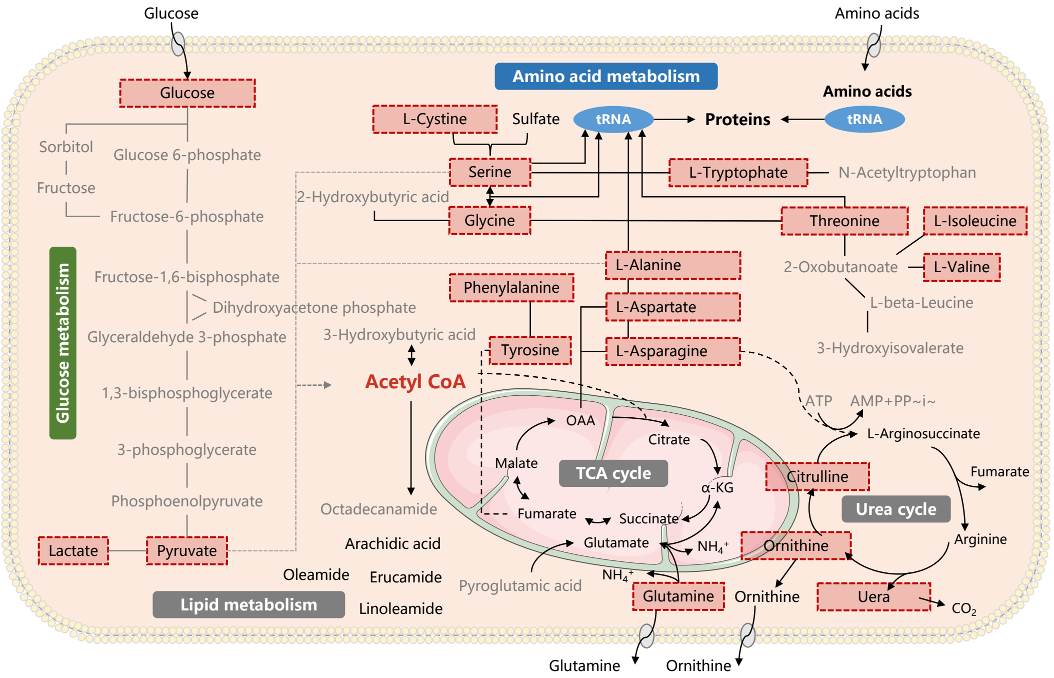

(a)

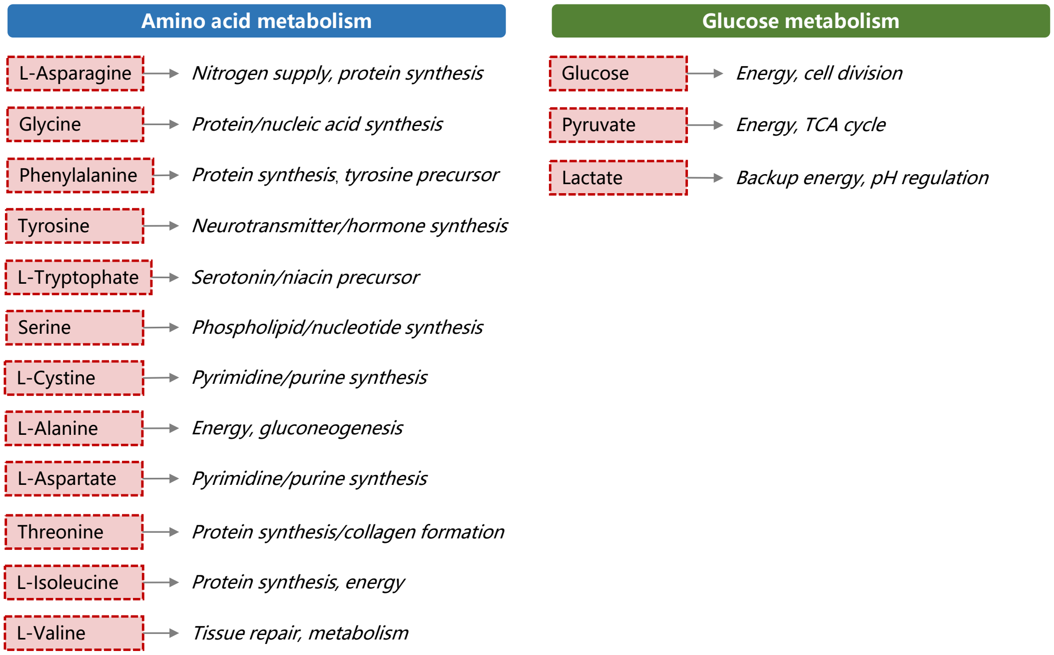

(b)

Supplementary Figure 3. (a) Glucose and Amino Acids Drive Macromolecule Synthesis. (The red dashed box indicates potential biomarkers within the glucose and amino acid metabolic pathways that be can used to assess embryonic developmental quality. tRNA: Transfer RNA; ATP: Adenosine Triphosphate; AMP: Adenosine Monophosphate; PP~i~: Pyrophosphate; OAA: Oxaloacetate; α-KG: α-Ketoglutarate.) (b) Functional mechanisms of multiple metabolites (e.g., glucose, amino acids) involved in the proposed research strategy during embryonic development. [2-8]

Reference

1. Liu J, Cai C, Wang Y, Liu Y, Huang L, Tian T, et al. A biomimetic plasmonic nanoreactor for reliable metabolite detection. Adv Sci (2020) 7:1903730. doi: 10.1002/advs.201903730
2. Deng S, Xu Y, Warden AR, Xu L, Duan X, He J, et al. Quantitative proteomics and metabolomics of culture medium from single human embryo reveal embryo quality-related multiomics biomarkers. Anal Chem (2024) 96:11832-11844. doi: 10.1021/acs.analchem.4c01494
3. Gao M, Huang J, Jiang X, Yuan Y, Pang H, Luo S, et al. Regulation of aerobic glycolysis to decelerate tumor proliferation by small molecule inhibitors targeting glucose transporters. Protein Cell (2020) 11:446-451. doi: 10.1007/s13238-020-00725-7
4. Yang W, Wang P, Cao P, Wang S, Yang Y, Su H, et al. Hypoxic in vitro culture reduces histone lactylation and impairs pre-implantation embryonic development in mice. Epigenetics Chromatin (2021) 14:57. doi: 10.1186/s13072-021-00431-6
5. Li T, Jin Y, Wu J, Ren Z. Beyond energy provider: multifunction of lipid droplets in embryonic development. Biol Res (2023) 56:38. doi: 10.1016/j.saa.2025.126475
6. He C, Huang S, Yi R, Zhang J, Yan N, Kong L, et al. Dynamic revelation of early development in mouse embryo via Raman spectroscopy. Spectrochim Acta A Mol Biomol Spectrosc (2025) 342:126475. doi: 10.1016/j.saa.2025.126475
7. Scalici E, Traver S, Molinari N, Mullet T, Monforte M, Vintejoux E, et al. Cell-free DNA in human follicular fluid as a biomarker of embryo quality. Hum Reprod (2014) 29:2661-2669. doi: 10.1093/humrep/deu238
8. Song X, Zhou Z, Liu J, Li J, Yu C, Zeh HJ, et al. Cytosolic cytochrome c represses ferroptosis. Cell Metab (2025) 37:1326-1343. doi: 10.1016/j.cmet.2025.03.014
